# Adult report of childhood imaginary companions and adversity relates to concurrent prodromal psychosis symptoms

**DOI:** 10.1016/j.psychres.2018.11.046

**Published:** 2019-01

**Authors:** Paige E. Davis, Lisa A.D. Webster, Charles Fernyhough, Kevin Ralston, Susanna Kola-Palmer, Helen J. Stain

**Affiliations:** a University of Huddersfield, Psychology Department, Queensgate, Huddersfield, HD1 3DH, United Kingdom; b Leeds Trinity University, Psychology Department, Brownberrie Ln., Leeds, LS18 5HD, United Kingdom; c Durham University, Psychology Department, Science Site, South Rd. Durham, DH1 3LE, United Kingdom; d York St. John University, School of Psychological and Social Sciences, Lord Mayor's Walk, York, YO31 7EX, United Kingdom

**Keywords:** Imaginary companions, Prodromal symptoms, Childhood adversity

## Abstract

Hallucination and dissociation have been found to be associated with imaginary friend play in childhood (CIC). Past studies have not investigated how this play relates to adult prodromal symptoms or how childhood adversity mediates the relationship. CIC play was examined in 278 participants, 18–24 years. CIC status predicted prodromal symptoms of hallucination only, whereas childhood adversity predicted all other symptoms. Mediation analysis found CIC's relation to hallucination symptoms was partially mediated by childhood adversity. Findings fit with views that CIC are a positive childhood experience which may convert to a negative developmental trajectory through the impact of childhood adversity.

## Highlights

• Childhood imaginary friend report predicted adult prodromal hallucination symptoms.
• Childhood imaginary friend report did not predict other adult prodromal symptoms.
• Childhood adversity report predicted all types of adult prodromal symptoms.
• Childhood adversity mediated imaginary friend prodromal hallucination symptoms.

## 1 Introduction

Childhood imaginary companion (CIC) play is a positive normative experience which has been found to relate to skills such as better theory of mind (Taylor and Carlson, 1997), emotional understanding (Giménez-Dasi et al., 2016) and mental state orientation (Davis et al., 2014) in children who create these entities. Furthermore, high-risk pre-adolescents with CICs have been found to have better coping skills and better positive adjustment than their peers (Taylor et al., 2010).

CICs have been argued to be a non-pathological indicator of early propensity to hallucinate (Pearson et al., 2001). Children with imaginary companions have been found to score higher on early dissociation scales (Carlson et al., 2008), and to hear more words in an ambiguous voice stimuli aimed at simulating auditory hallucination-like experiences (Fernyhough et al., 2007).

There have been studies finding adult that dissociative experiences relate to CIC play (Dierker et al., 1995), but no studies have investigated whether this play predicts later adult hallucination experiences in the form of prodromal symptoms. Our aim was to relate adult university students’ report of CIC play to the reporting of different prodromal symptoms (hallucination, perceptual abnormalities, negative symptoms), taking into account childhood adverse experiences. Childhood adversity paired with high imagination has been hypothesised to be a predisposing factor in dissociation as well as predictive of prodromal symptoms in adulthood (Carlson et al., 2008, Varese et al., 2012).

## 2 Method

Data were gathered as part of an online survey conducted at a UK Higher Education Institution examining the social and emotional wellbeing of university students (SoWise).

This study reports on a subset of the measures from the SoWise survey using a purposive sub-sample of participants for analysis. Participants over the age of 24 were excluded for two reasons: 1) prodromal symptoms are known to occur from the onset of puberty to the early 20s (Trotman et al., 2013) and 2) recall of imaginary companions is likely to be compromised with age. The final sample consisted of 278 (196 females) students aged 18–24 years (*M = *20.38, *SD = *1.56). Sociodemographic data included age, gender, and parental socioeconomic status. Some participants did not complete all sections; missingness was treated by deletion.

CIC engagement was determined by the Imaginary Companion Questionnaire (Taylor and Carlson, 1997), defining CICs as follows: *An imaginary friend can be classified as completely invisible, OR a doll or toy that you had given a personality to and played with for over 3 months*. Participants were asked if they remembered having a CIC and if so what characteristics they exhibited (e.g. age, gender, humour). All participants reporting a CIC completed the qualitative questions about their companion. Qualitative questions were used to determine whether CICs were invisible or personified. If participants left the initial CIC question blank they were not considered in the analyses.

The screening measure for prodromal symptoms of psychosis was the Prodromal Questionaire-16 (PQ-16) (Loewy et al., 2005). This self-report measure includes 16 true/false items, nine assessing hallucinations/perceptual abnormalities, five assessing unusual thought content/delusional ideas/paranoia, and two assessing negative symptoms of anxiety and depression. Higher scores on this measure indicated more symptoms/greater severity.

Finally, the Childhood Experience of Care and Abuse Questionnaire (CECA-Q) (Bifulco et al., 1994) was used as a retrospective self-report measure of childhood adversity before the age of 17. The scale consists of questions on parental loss, neglect, apathy, and physical/sexual abuse. An adverse experiences subscale was created using questions from the CECA-Q. Reports of physical and sexual abuse severity scores from 0–9 for physical abuse and 0–10 for sexual abuse were consolidated into a 0–4 scale (based on Bifulco et al., 2002) indicating severity of childhood adverse experiences (marked, moderate, mild, little or none). The 0–4 score was collapsed to a dichotomous variable representing marked/moderate abuse, or mild/little/no abuse reported in order to determine severe from non-severe experiences (Bifulco et al., 2002). Higher scores indicated greater severity for this measure.

## 3 Results

Of the 278 students, 224 reported on CIC status. 62 (22%) reported CIC play, with one removed because the CIC was described as a result of psychosis. There were no age effects, *t* (222) = −0.88, *p = *.381, but females *X*^2^ (*N* = 224) = 4.96, *p* = .026 reported significantly more CIC play.

The PQ-16 was completed by 218 (56 male) participants, reporting a range of 0–15 symptoms (*M = *4.53, *SD = *3.59). No gender, *t* (216) = −0.54, *p = *.590, or age, *r = 0*.07, *p = *.331, differences were found.

The CECA-Q was completed by 225 (60 male) participants. No gender, *X*^2^ (*N* = 225) = 1.64, *p* = .201, or age, *t* (223) = −0.72, *p = *.475, differences were found for this variable.

The relationship between CIC status, prodromal symptoms, and adverse childhood experiences was measured using three Poisson regressions with 1) hallucinations/perceptual abnormality, 2) unusual thought content/delusional ideas/paranoia, and 3) negative prodromal symptoms reported as the outcome variables. CIC status and high/low adversity in childhood were entered as predictor variables. Gender and socioeconomic status were covariates. An interaction term was also entered.

Those reporting no CIC (NIC) in childhood in the hallucination model reported 53% less prodromal hallucination symptoms in comparison to the CIC group, Exp (B) 0.473 (95% CI, −1.216 to −0.283); this was a significant predictor, *p = *.002. Those reporting low adversity in the hallucination model also reported 58% less prodromal symptoms than those with high adversity scores, Exp (B) 0.423 (95% CI, −1.194 to −0.528); this was also significant, *p *< .001. Furthermore there was a significant interaction, Exp (B) 1.935 (95% CI, 0.137 to 1.183); *p *= .013. Those reporting both CIC and high adversity reported 94% more prodromal symptoms.

A mediation analysis was run using Stata-15 and the PARAMED module using the bootstrap method to estimate bias-corrected confidence intervals. The relationship between CIC status and hallucination symptoms was mediated by childhood adversity where the total effect was significant (Estimate = 1.36, CI, 1.11 to 1.68) *p = .*003, as well as the natural direct effect (Estimate = 1.25, CI, 1.02 to 1.54) *p = *.032, and the natural indirect effect (Estimate = 1.09, CI, 1.02–1.16) *p = *.007.

There were no main effects of CIC status in the model predicting unusual thought content symptoms, *p* = .088. Those reporting low adversity scores reported 40% less prodromal symptoms than those reporting high adversity, Exp (B) 0.601 (95% CI, −0.914 to −0.104); this was significant, *p *= .017. No significant interaction was found, *p = *.338.

In the third regression looking at negative symptoms, there was no significant contribution of CIC status, *p* = .306; however those reporting low adversity reported 47% less prodromal symptoms than those with high adversity in childhood, Exp (B) 0.527 (95% CI, −1.172 to −0.111), *p *= .018. There was no interaction, *p = *.333.

Adults with a CIC were more likely to report high adversity, *X*^2^ (N = 224) = 8.32, *p* = .004. See Table 1 for descriptive statistics for all variables.

**Table 1:** Descriptive statistics for all variables according to childhood imaginary companion status: means followed by standard deviations.

Variables		*N*	CIC	*N*	NIC	*N*	Total
Age (years)		62	20.23 (1.51)	162	20.43 (1.60)	224	20.38 (1.57)
Gender	Female	52	23%	112	50%	164	73%
	Male	10	4%	50	22%	60	27%
PQ-16		60	5.35 (4.05)	158	4.22 (3.37)	218	4.53 (3.59)
	Hallucination	60	2.47 (2.27)	158	1.79 (1.97)	218	1.98 (2.10)
	Unusual thought	61	1.87 (1.52)	158	1.51 (1.33)	219	1.61 (1.40)
	Negative	61	1.03 (0.82)	158	0.92 (0.80)	219	0.95 (0.80)
CECA-Q	Low adversity	49	22%	150	67%	199	89%
	High adversity	13	6%	12	5%	25	11%
SES	Under £10k	11	18%	29	18%	48	18%
	£10k−25k	26	42%	64	40%	110	41%
	Over 25k	25	40%	68	42%	112	42%

*Note There are missing data for some columns. The N reflects the number of participants that chose to answer each question.

## 4 Discussion

Reporting concurrent prodromal symptoms of hallucination/perceptual abnormality was associated with self-report of CICs. CIC status did not predict report of unusual thought content or negative symptoms. Childhood adversity, however, related to all three types of prodromal symptoms and partially mediated the relationship between CICs and hallucination/perceptual symptoms.

These findings are in line with previous research suggesting that having a CIC should be considered a form of non-pathological hallucination-like-experience (Fernyhough et al., 2007) that may impact adult experiences (Burbach et al., 2014). Although CIC status was predictive of prodromal symptom report of hallucination/perceptual abnormality, childhood adversity predicted all three components of the prodromal symptoms, and both mediated and moderated the hallucination/perceptual abnormality symptom relationship between CIC status and prodromal symptom report. This is consistent with literature on trauma and its relationship with prodromal symptoms (Thompson et al., 2009). Because the sample was typical, no predictions could be made about pathology. Furthermore, the scores on the PQ-16 were lower than they would be for a clinical sample (Savill et al., 2018). Future research, therefore, might focus on how life events mediate the relationship between pathological and non-pathological hallucinations, as CICs are known to be a positive form of play that relate to better social cognition (Giménez-Dasí et al., 2016).

Several interpretations of the results are possible. Firstly, children's experiences with typical hallucination-like experiences in the form of interacting with a CIC may become prone to experience non-clinical prodromal symptoms of hallucination with the addition of life stressors. Secondly, children with CICs may become accustomed to speaking about imaginary invisible beings or voices throughout their development, and would be more likely to report these experiences than their peers.

Limitations of the survey design were mitigated through the use of reliable and valid measures appropriate to the population. It would be useful to incorporate longitudinal design to find out more about the prodromal symptom trajectory of CIC individuals from childhood to adulthood. The regressions did show dispersion in the sample as well as having an unequal sample size; however these parametric tests have been shown to be robust against violations of assumptions (Tabachnick and Fidell, 2013).

In summary, this research is consistent with the developmental perspective of the continuum of hallucination from pathological to non-pathological, and supports the notion that CICs may be a form of hallucination-like-experience which, particularly when combined with adversity, may influence the trajectory of non-clinical prodromal hallucination report.

## Ethical statement

The authors assert that all procedures contributing to this work comply with the ethical standards of the relevant national and institutional committees on human experimentation and with the Helsinki Declaration of 1975, as revised in 2008. All procedures involving human subjects were approved by the university ethics review board.

## Consent statement

Written consent was obtained from all subjects.

## Declaration of interests

Dr. Davis: None, has nothing to disclose, Dr. Webster: None, has nothing to disclose, Dr. Fernyhough: None, has nothing to disclose, Dr. Ralston: None, has nothing to disclose, Dr. Kola-Palmer: None, has nothing to disclose, Professor. Stain: None, has nothing to disclose.
